# Supplementary material for: Visual analysis of mass cytometry data by hierarchical stochastic neighbour embedding reveals rare cell types
Source: Nat Commun. 2017 Nov 23;8:1740. doi: 10.1038/s41467-017-01689-9 (PMC5700955; doi:10.1038/s41467-017-01689-9)
Supplement: Supplementary file 1 — Supplementary Information [file 41467_2017_1689_MOESM1_ESM.pdf]

Supplementary Note 1. Cytosplore^{+HSNE} is reproducible and robust

Cytosplore^{+HSNE} allows significant user interaction during the exploration of the HSNE hierarchy, where the embedding visualizations and integrated clustering provide strong guidance. Independent explorations of the 5.2 million dataset, following the same zooming-in strategy are shown in **Supplementary Figure 5**. While the embeddings slightly vary at all levels, (mostly in rotation and reflection of the map), the same high level structure is found in all explorations. The robust separation of these structures guides the user in the selection and zooming-in process, resulting in highly similar embeddings down to the data level.

Focusing on separate regions of the data and interactively zooming into these separately provides significantly more detail than is possible by direct dimensionality reduction or clustering of the complete dataset (**Figs. 3** and **4**). However, Cytosplore^{+HSNE} does provide the possibility to visualize the complete dataset at the data level (**Supplementary Fig. 1a**). A dataset consisting of 1 million cells created by randomly sampling the 5.2 million cell dataset presented in the main text and three smaller ones derived from this were analysed with HSNE and t-SNE resulting in highly similar embeddings (**Supplementary Fig. 1a**).

Supplementary Figure 1b shows the robustness of HSNE with regard to downsampling as well as the superiority of the HSNE data reduction towards the overview level, compared to random downsampling. Here the embeddings within each column are similar, indicating that HSNE captures similar features even with downsampled data. However, detail increases with growing data sizes even if the number of landmarks are comparable between datasets. Thus the HSNE hierarchy preserves the non-linear structures in the data when reducing the data for visualization at the more abstract levels, while these structures can be lost during random downsampling.

The difference in detail is especially striking when comparing the complete HSNE hierarchy of 1 million cells (**Supplementary Fig. 1b**, top row) to the t-SNE embeddings of randomly sampled datasets of similar sizes as the HSNE levels (**Supplementary Fig. 1a**, bottom row).

Supplementary Note 2. Millions of cells cause performance issues and overcrowding in t-SNE

Although feasible with a strong computational infrastructure, t-SNE suffers from several problems when analyzing datasets exceeding hundreds of thousands of cells. Three main parameters influence

the result of a t-SNE embedding: the number of iterations for the gradient descent *i*, perplexity *p* and theta *t* (the latter only for BH-SNE). Cytobank provides a brief analysis of the parameters[a] that shows diminishing returns for p and t, beyond certain values, which can sensibly be used as defaults and do not significantly change with the input data size. In contrast, i needs to be adjusted with increasing data sizes. We show that the commonly used default value of i=1,000 is not enough to properly embed millions of cells (**Supplementary Fig. 2**). All embeddings were created using A-tSNE, implemented in Cytosplore, using the default parameters of p=30 and t=0.01. **Supplementary Figure 2a-c** show embeddings of 1 million, 2 million and 5 million cells, respectively, randomly sampled from the 5.2 million cell dataset presented in the main text after 1,000 iterations. Computation time for the embeddings were (**a**) 5.5 h, (**b**) 13 h, and (**c**) 54 h. **Supplementary Figure 2d-f** show the same embeddings after 4,000 further iterations. Total computation time for the embeddings were (**d**) 19.5 h, (**e**) 45.5 h, and (**f**) 252 h.

While **Supplementary Figure 2a** seems to provide a good separation for some high level clusters **Supplementary Figure 2b** and **c** show typical artifacts of a non-converged embedding, i.e. the cells concentrate strongly in the center of the visualization, often forming a cross shape along the two axes as is clearly visible in the density plots.

All embeddings evolved significantly after 4,000 additional iterations (**Supplementary Fig. 2d-f**), indicating that 1,000 iterations are not enough to fully converge for these large data sizes. Even after 5,000 iterations and 252 h of computation **Supplementary Figure 2f** still shows similar artifacts.

Another problem of computing t-SNE for such large datasets is overcrowding. All embeddings show signs of overcrowding. Only large scale neighborhoods can be identified in **Supplementary Figure 2d**, while structure within these neighborhoods is hard to identify due to the large number of cells, even in the density plot. Also, in **Supplementary Figure 2e** and **f** some 'color smear' is present in the single-cell plots indicating that local neighborhoods were not resolved properly by the t-SNE algorithm. Intuitively, t-SNE accounts for small neighborhoods. By increasing the size of the input data local neighborhoods will often become less strongly connected and can tear, resulting in the displacement of cells in the plot. These effects might be reduced by increasing the perplexity value[b]. Increasing p will help in the separation of high level clusters, however, at the cost of intracluster separation, as there will be less visual space for each cluster. A detailed analysis of the neighborhood conservation of different dimensionality reduction techniques, including t-SNE, can be found in our previous work[13].

---

[a] https://support.cytobank.org/hc/en-us/articles/206439707-How-to-Configure-and-Run-a-viSNE-Analysis#iterations

[b] http://blog.cytobank.org/2017/01/17/fine-tune-visne-to-get-the-most-of-your-single-cell-data-analysis/

Supplementary Note 3. Cytosplore^{+HSNE} offers advantages over current scalable single-cell analysis methods

We investigated the generalizability as well the scalability of Cytosplore^{+HSNE} by comparison to two other state-of-the-art scalable single-cell analysis methods and accompanying public datasets (Phenograph and VorteX). Both techniques use a clustering method followed by visualization of the generated clusters.

Phenograph achieves this by the Louvain community detection method for partitioning of the kNN graph, followed by a t-SNE embedding of the communities based on their median values. The resulting embedding places the communities in a global context, but cannot display the details of the single-cell complexity within the communities. Using Cytosplore^{+HSNE} we were able to reproduce the clusters of the Phenograph bone marrow dataset, consisting of 15 million cells, after 3.5 hours of computation, compared to 40 hours with the Phenograph algorithm (clustering per individual samples) on the same computer. Also, Cytosplore^{+HSNE} only required 29 minutes to compute the 5.2 million cell gastrointestinal dataset, while Phenograph required 4 hours. In addition to the significantly faster computation, Cytosplore^{+HSNE} provides the distinct advantage of visualizing all cells and intracluster heterogeneity at subsequent levels of detail (**Supplementary Fig. 7**).

VorteX first clusters the data using the X-shift algorithm, and then visualizes the result by random sampling of cells from the clusters for visualization in a single-cell force-directed layout. The sampling is necessary, as the force-directed layout can computationally handle 30,000 cells only. Therefore, the resulting single-cell visualization shows only 3.6 % of the original dataset. Although the technique allows for more detailed cellular visualization compared to Phenograph, a time-consuming second computation is required for every additional analysis on individual immune lineages. In a direct comparison Cytosplore^{+HSNE} recapitulated the murine bone marrow clusters at the second level of a 4 level hierarchy in 4 minutes while VorteX required 22 hours (**Supplementary Fig. 8a,b**). In addition, by applying the zooming-in approach, we obtained the single-cell details for the plasmacytoid dendritic cell lineage within seconds (**Supplementary Fig. 8c**). Finally, VorteX failed computing the 5.2 million cell gastrointestinal dataset within 3 days of clustering (regardless of using Euclidian or Angular distance).

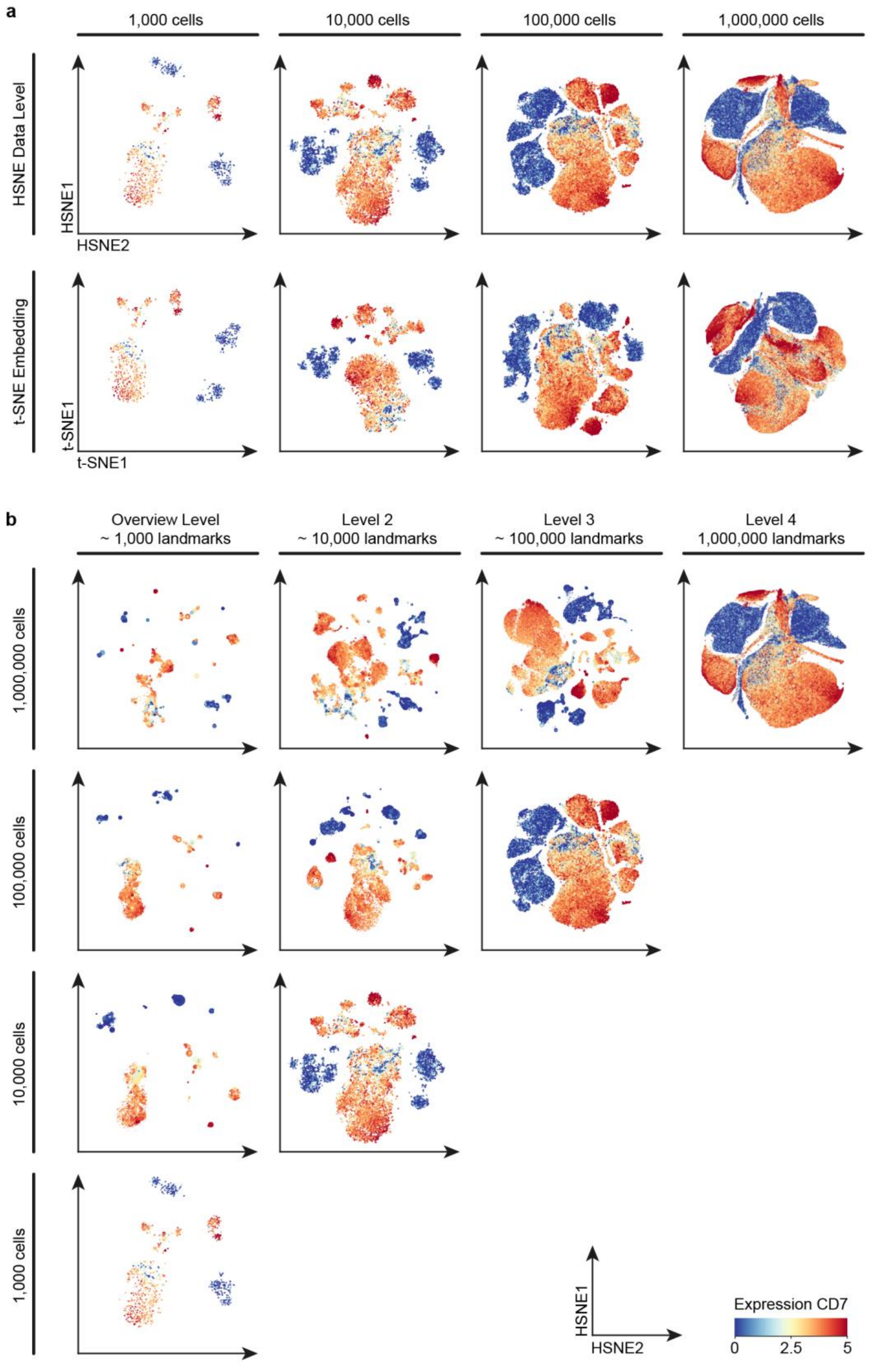

Supplementary Figure 1 Comparison of robustness with regard to downsampling between t-SNE and HSNE.

(**a**) Comparison of t-SNE (bottom row) and HSNE (top row) data level embeddings for datasets of different sizes (columns). First, 1 million cells were randomly sampled from the 5.2 million cell dataset, the smaller datasets were then created by randomly sampling the next largest one. All plots were created after 1,000 iterations. The 1 million cell embeddings were not fully converged. Color indicates CD7 expression.

(**b**) Robustness of the HSNE hierarchy with regard to downsampling. Each row shows the datasets as described above. Embeddings for the complete hierarchy of log10(N / 100) levels, with N being the number of cells, are shown in the columns. Color as in panel a. Numbers of landmarks are approximated, indicating a reduction of one order of magnitude per level. In all columns the amount of detail increases towards the top (larger datasets), even though all embeddings in a column consist of roughly the same number of points. This implies that the preservation of non-linear neighborhoods by HSNE conserves structure that is lost by random downsampling.

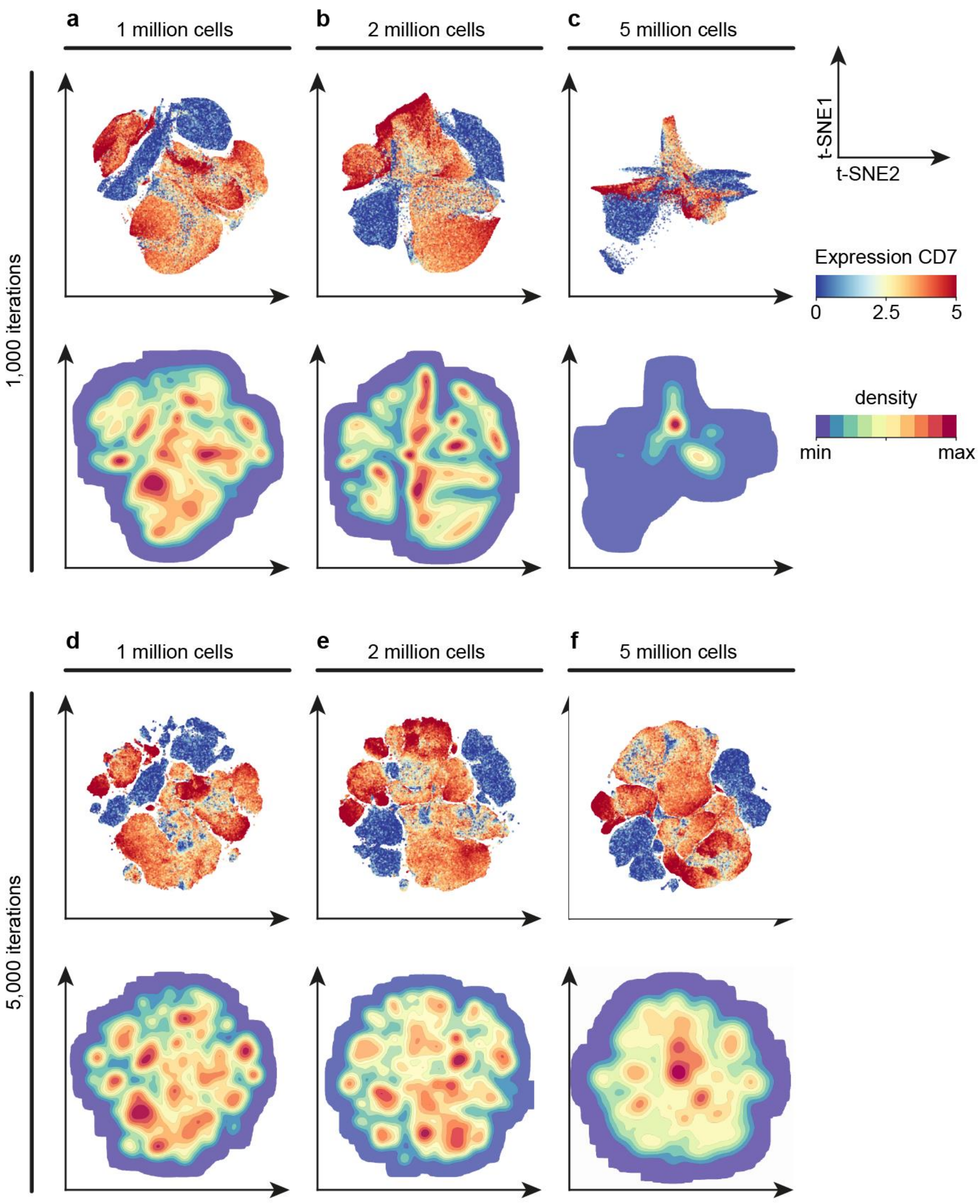

g

iterations	1 million cells (memory used: 16GB)	2 million cells (memory used: 38GB)	5 million cells (memory used: 99GB)
1,000	5.5 h	13 h	54 h
5,000	19.5 h	45.5 h	252 h

Supplementary Figure 2 t-SNE embeddings of millions of cells show overcrowding and artifacts caused by insufficient optimization.

(**a-c**) Single-cell (top row) and density-based (bottom row) visualizations of t-SNE embeddings of (**a**) 1, (**b**) 2 and (**c**) 5 million cells, respectively, after 1,000 iterations, the standard setting used in many t-SNE applications. Color in the single-cell visualization corresponds to the CD7 marker expression; in the density visualization to the cell density in the t-SNE plot.

(**d-f**) The same embeddings, consisting of (**d**) 1, (**e**) 2 and (**f**) 5 million cells, respectively, after 4,000 additional iterations, resulting in a total of 5,000 iterations. Colors as above.

(**g**) Computation times for the different t-SNE computations.

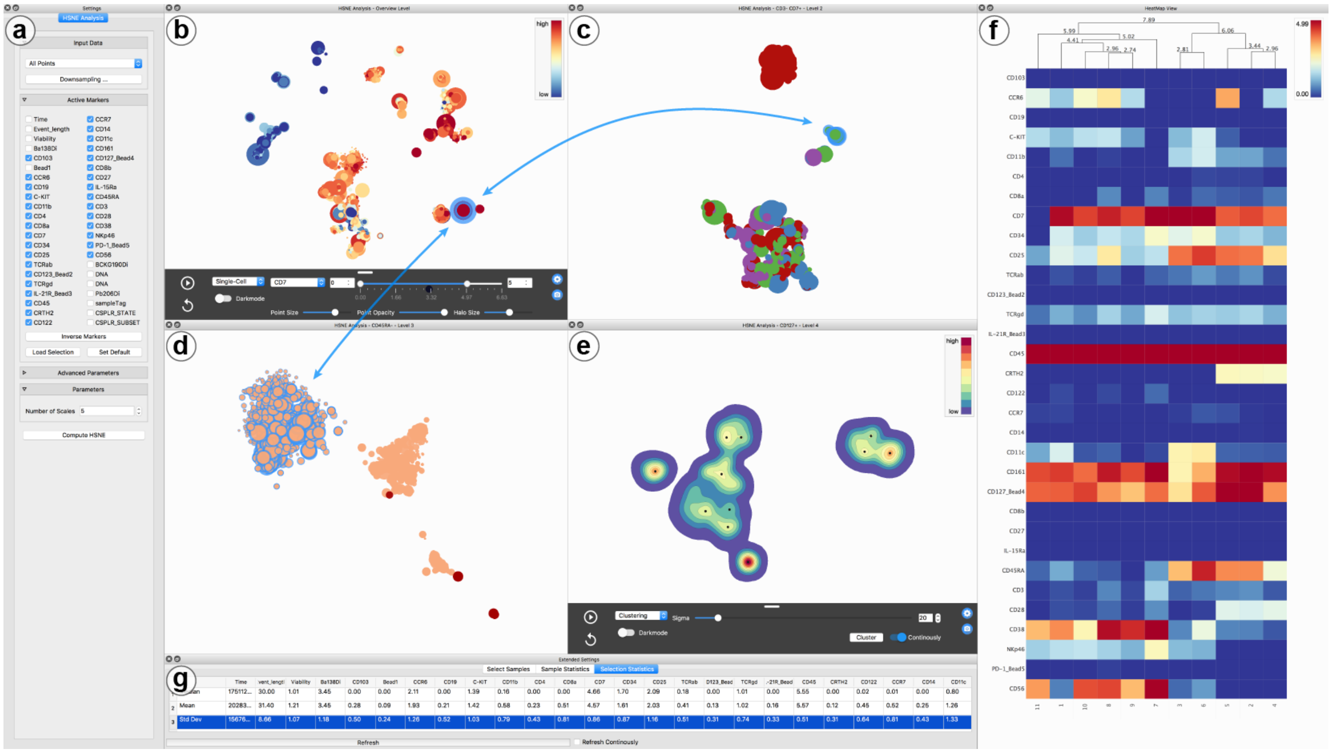

Supplementary Figure 3 The Cytosplore^{+HSNE} software.
(a) Settings panel for the HSNE analysis.
(b-e) Zoom into the Innate Lymphocytes as shown in Figure 2 and Supplementary Figure 3. **(b)** overview level, **(c)** level 2, **(d)** level 3, **(e)** level 4. Color shows; **(b)** CD7 marker expression, **(c)** clinical features, **(d)** tissue origin, **(e)** cell density. A selection in panel d is highlighted in panel b,c, and d by blue halos around circles and arrows. Note, arrows added for clarity only and are not part of the software.
(f) heatmap visualization of the median values of the clusters generated by GMS clustering based on the density visualization in panel e. Color shows marker expression.
(g) Statistics of the selection shown in panel b-d.

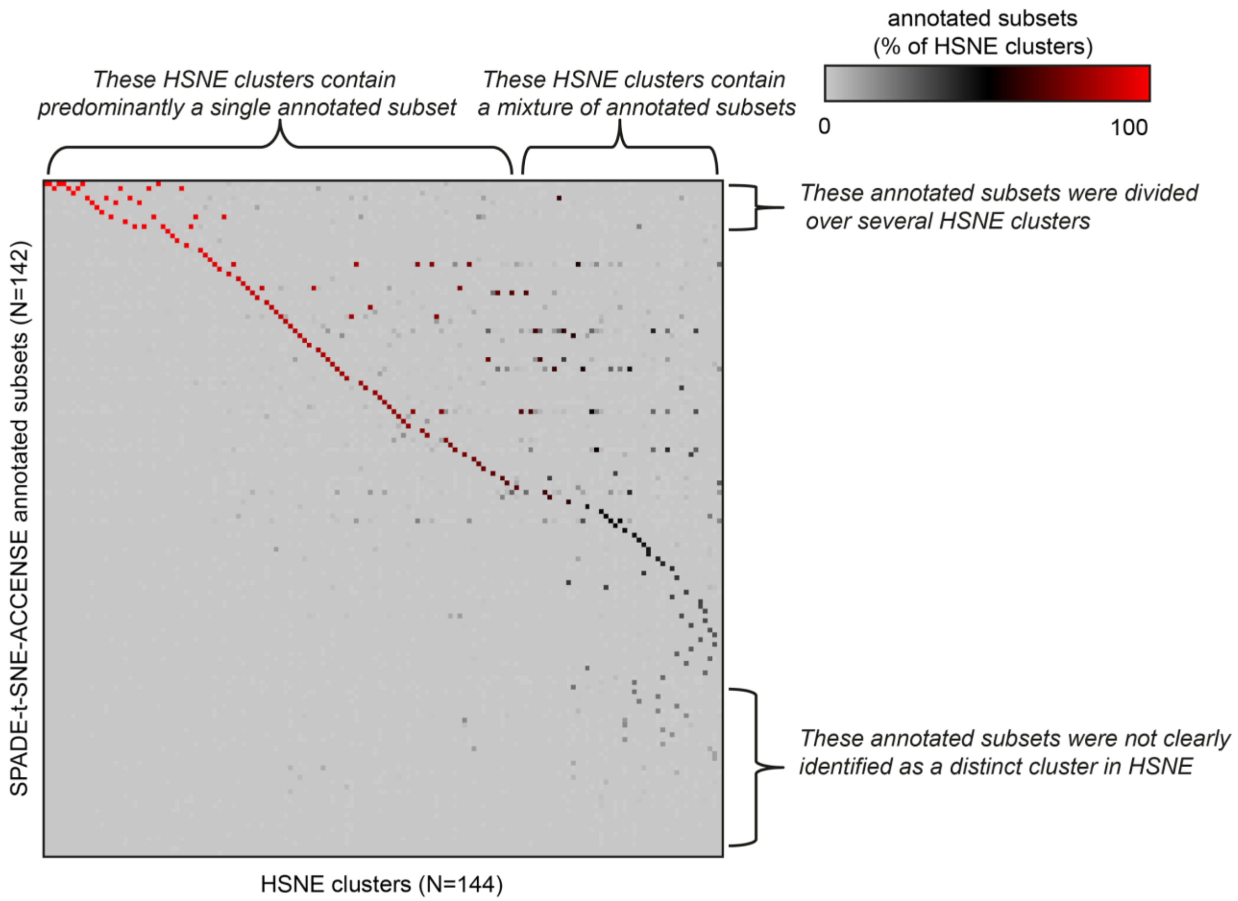

Supplementary Figure 4 Comparisons of cellular composition of the clusters identified with Cytosplore^{+HSNE} with the previously annotated subsets using the SPADE-t-SNE-ACCENSE method.

Rows indicate the individual SPADE-t-SNE-ACCENSE annotated subsets (N = 142) identified in the previous study[14] (N = 142) and columns indicate the individual clusters identified with Cytosplore^{+HSNE} (N = 144) of the same 1.1 million cells from the gastrointestinal dataset. Color indicates the fraction of the cluster containing cells assigned to a single subset as annotated with SPADE-t-SNE-ACCENSE.

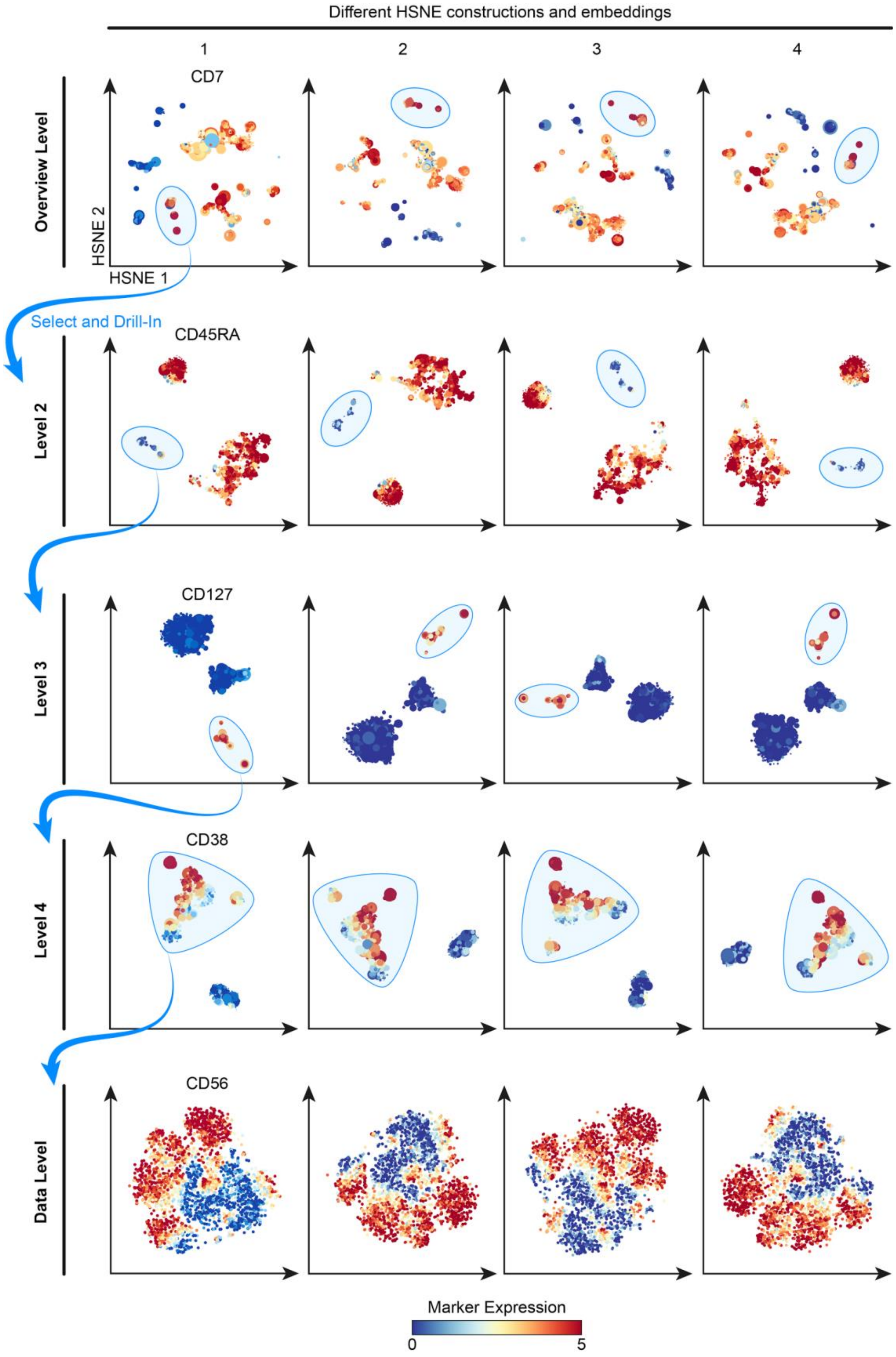

Supplementary Figure 5 Reproducibility of the hierarchy and the embeddings.

Four independent Cytosplore+HSNE analyses are shown (columns) reproducing the hierarchy construction and exploration of the data with the same zooming-in strategy (blue encirclements). Color-coding indicates arcsin5-transformed marker expression.

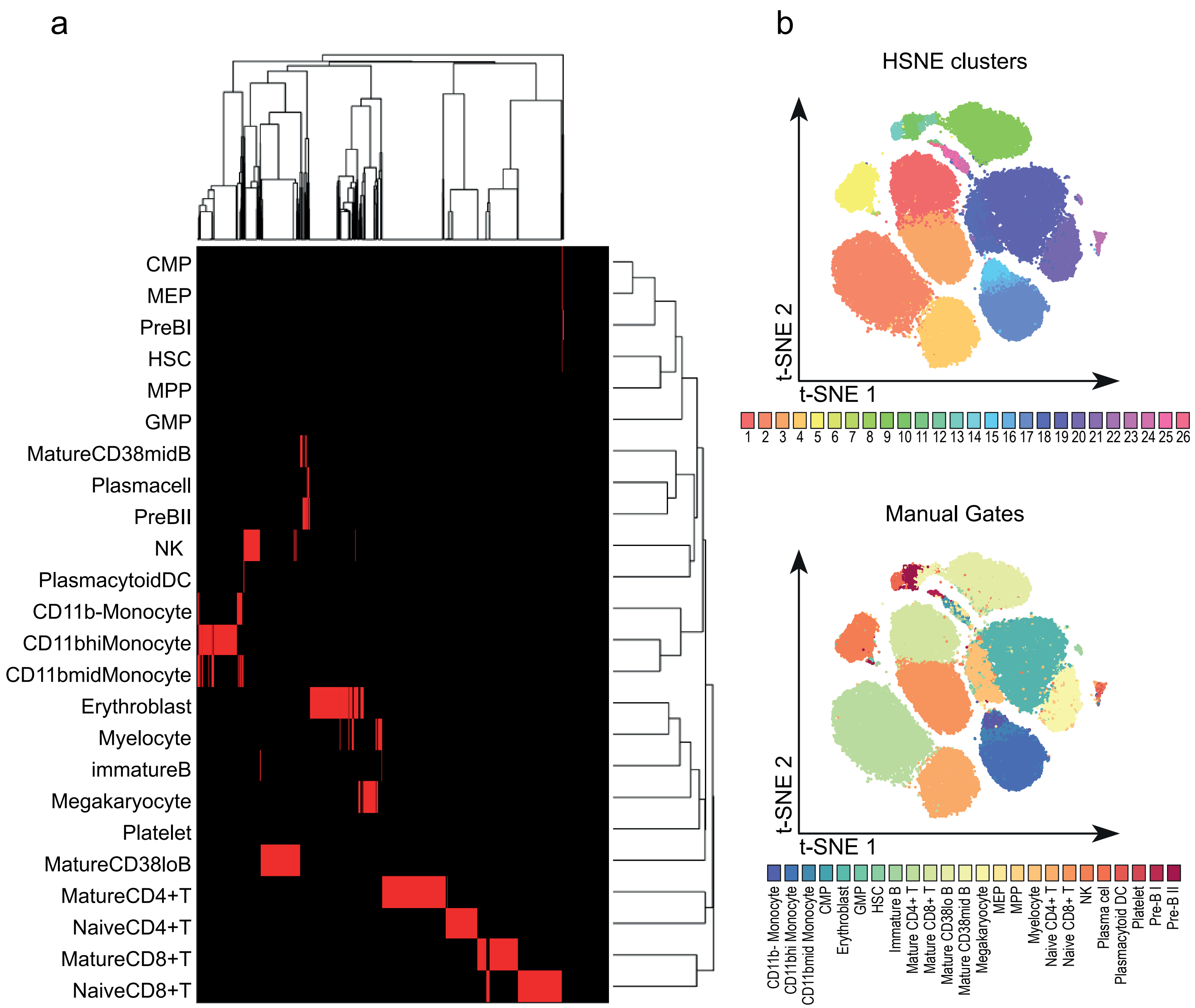

Supplementary Figure 6 Cytosplore^{+HSNE} analysis of the manual gated bone marrow benchmark dataset.
(**a**) Heatmap depicting the distributions of the landmarks in the overview level (N=2,632; 1st hierarchical level of 3 in total) across the manual gated subsets of the bone marrow benchmark dataset[27] (N=81,747 cells) and hierarchical clustering thereof. (**b**) t-SNE embedding of the bone marrow benchmark dataset color-coded for cell clusters identified with Cytosplore^{+HSNE} (top panel) or by cell type assignments established by manual gating (bottom panel).

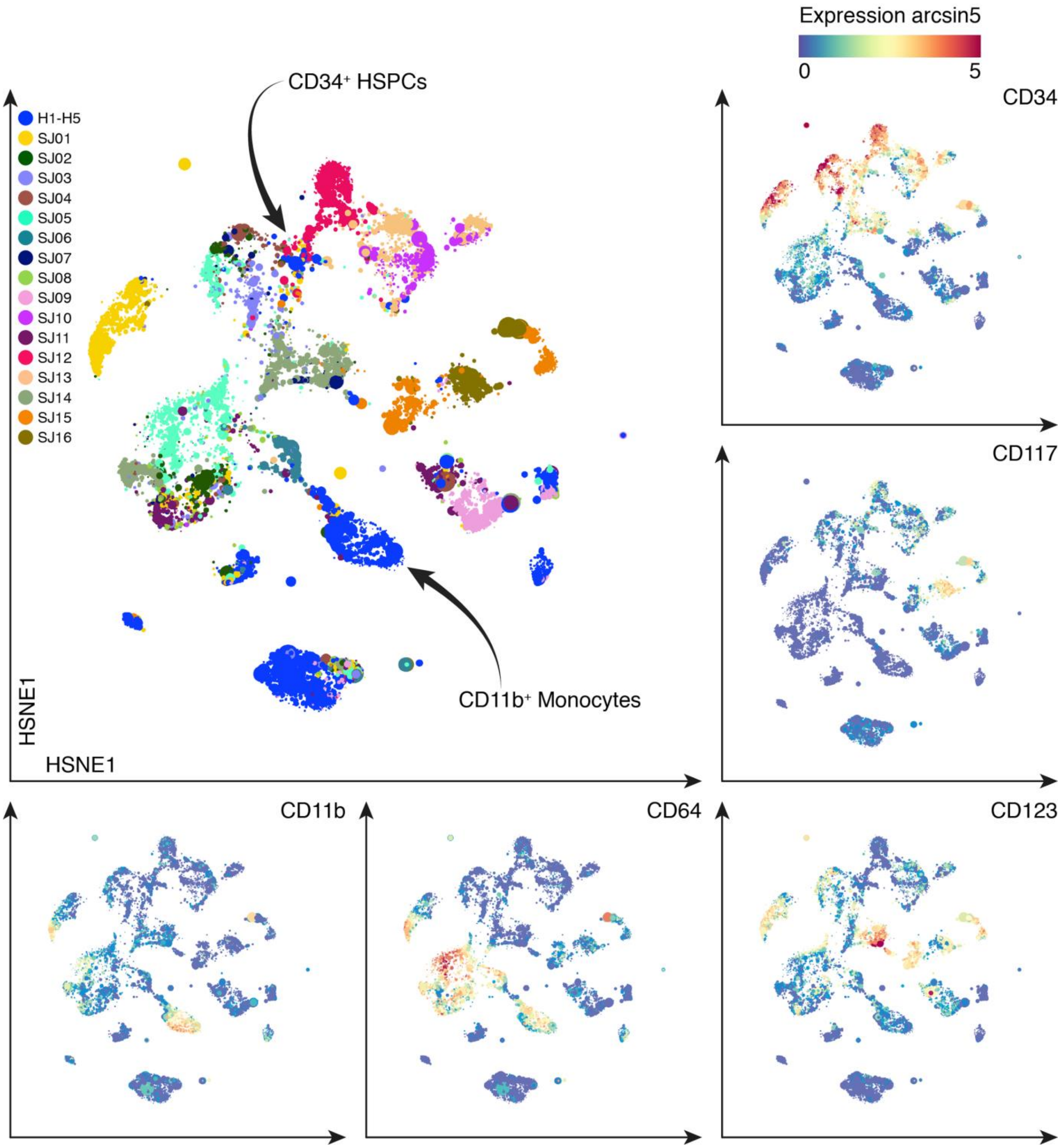

Supplementary Figure 7 Cytosplore^{+HSNE} analysis of the Phenograph bone marrow dataset.

Cytosplore^{+HSNE} embeddings of the full 15.0 million cells of the Phenograph human bone marrow dataset (overview level of a 5 level hierarchy). Color coding of main panel (top left) by patient identity. In additional panels, color coding indicates arcsin5-transformed marker expression. The above shows a comparison with Figure 3 of the original study[4].

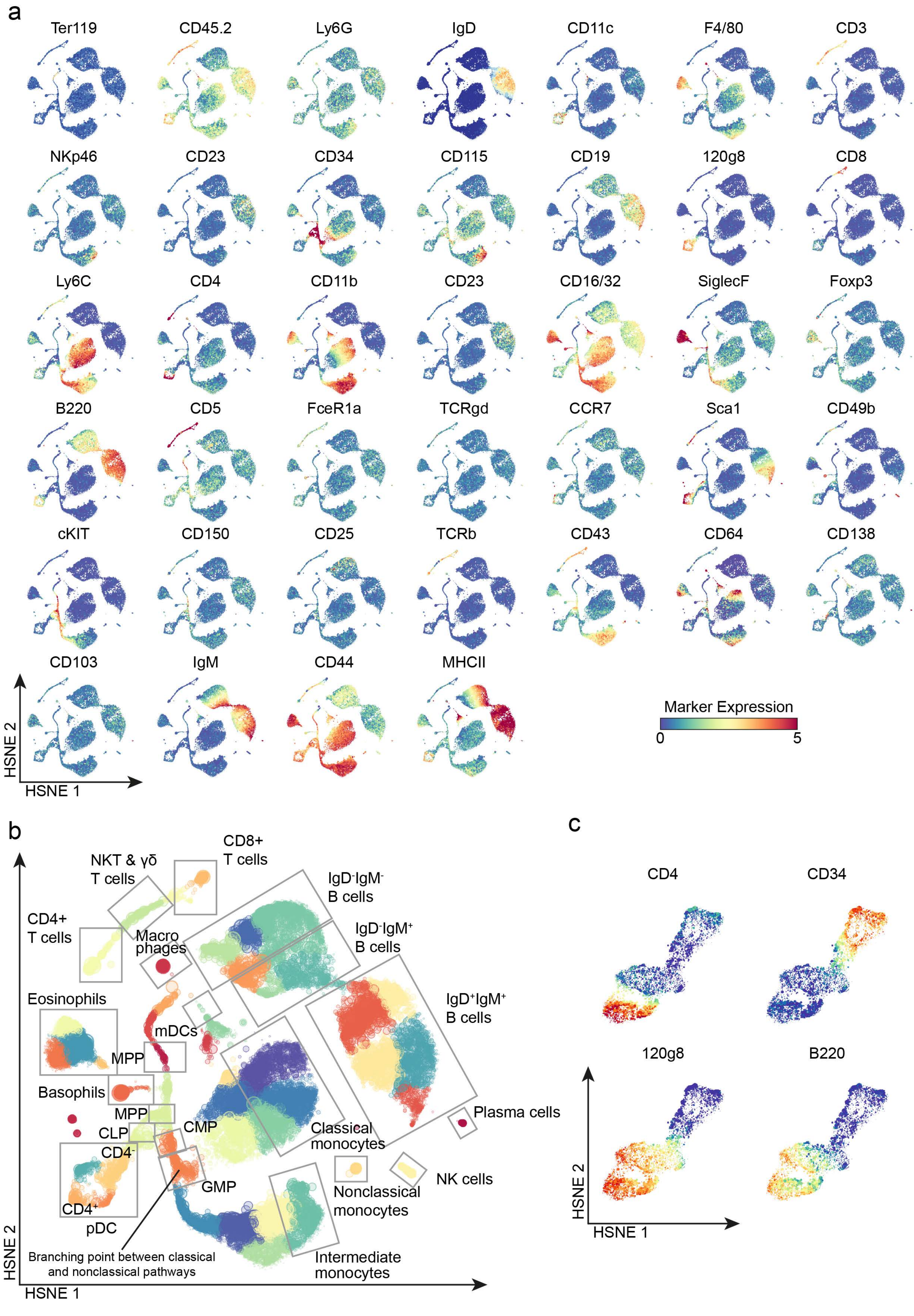
a
Ter119
CD45.2
Ly6G
IgD
CD11c
F4/80
CD3
NKp46
CD23
CD34
CD115
CD19
120g8
CD8
Ly6C
CD4
CD11b
CD23
CD16/32
SiglecF
Foxp3
B220
CD5
FceR1a
TCRgd
CCR7
Sca1
CD49b
cKIT
CD150
CD25
TCRb
CD43
CD64
CD138
CD103
IgM
CD44
MHCII
HSNE 2
HSNE 1
Marker Expression
0
5
b
CD8+ T cells
NKT & γδ T cells
IgD-IgM- B cells
CD4+ T cells
Macro phages
IgD-IgM+ B cells
Eosinophils
IgD+IgM+ B cells
mDCs
MPP
Basophils
Plasma cells
MPP
CLP
CMP
Classical monocytes
CD4-
CD4+
pDC
GMP
Nonclassical monocytes
NK cells
Branching point between classical and nonclassical pathways
Intermediate monocytes
HSNE 2
HSNE 1
c
CD4
CD34
120g8
B220
HSNE 2
HSNE 1

Supplementary Figure 8 Cytosplore^{+HSNE} analysis of the VorteX bone marrow dataset.

(**a**) Cytosplore^{+HSNE} embeddings of the full 0.8 million cells of the VorteX mouse bone marrow dataset (2nd hierarchical level of 4 in total). Color coding indicates arcsin5-transformed marker expression. (**b**) Embedding as in panel a. Color coded for 50 clusters identified with Cytosplore^{+HSNE}. Shaded boxes show locations of hand-gated cell populations. (**c**) Embeddings of zoomed-in populations related to pDC development (3rd hierarchical level of 4 in total). The above shows a comparison with Figure 2 of the original study[5].

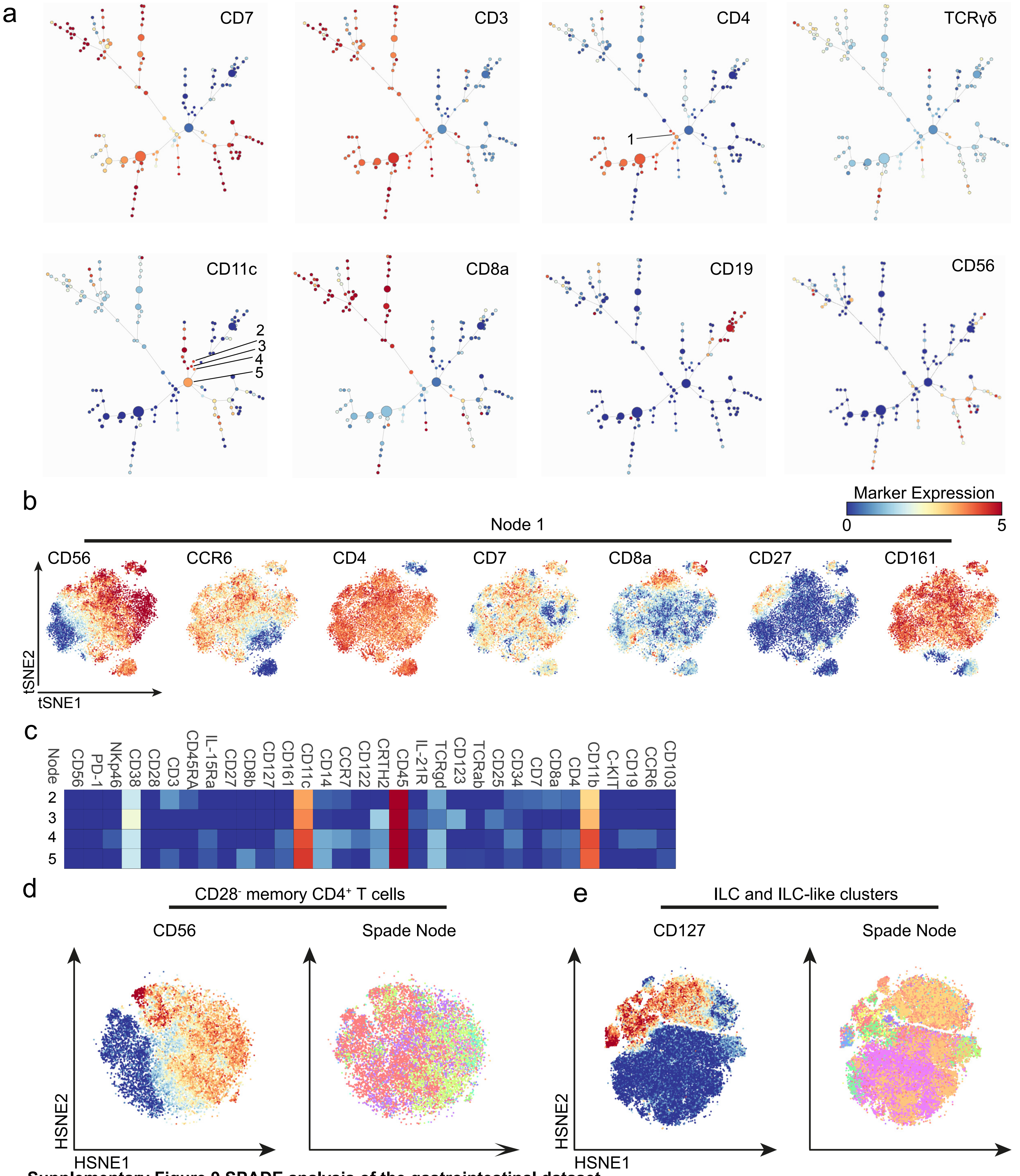

Supplementary Figure 9 SPADE analysis of the gastrointestinal dataset

The 5.2 million cell gastrointestinal dataset was analyzed by SPADE using 10% target events for density-based downsampling and 142 target nodes as settings. (**a**) SPADE tree colored with arcsin5-transformed marker expression as indicated. Size of the nodes represents cell size. Five nodes are indicated for further analysis. (b) t-SNE embedding of node 1 (panel a) at single-cell resolution. Color of cells as in panel a. (**c**) A heatmap summary of median expression values of cell markers expressed by nodes 2-5 (panel a). Coloring as in panel a. (**d**) HSNE data level of CD28- memory CD4+ T cells after clustering with SPADE. Colors indicate marker expression (left) and SPADE node identity (right) (**e**) HSNE data level of ILC and ILC-like clusters after clustering with SPADE.
