# Supplementary material for: Visual analysis of mass cytometry data by hierarchical stochastic neighbour embedding reveals rare cell types
Source: Nat Commun. 2017 Nov 23;8:1740. doi: 10.1038/s41467-017-01689-9 (PMC5700955; doi:10.1038/s41467-017-01689-9)
Supplement: Supplementary file 3 — Description of Additional Supplementary Files [file 41467_2017_1689_MOESM3_ESM.pdf]

Description of Supplementary Files

File name: Supplementary Movie 1
Description: Demonstration of a typical Cytosplore^{+HSNE} analysis. The video demonstrates the features of Cytosplore^{+HSNE} by example of an exploration of the gastrointestinal dataset on 5.2 million cells. The exploration starts with the computation of the hierarchy on the full dataset, without downsampling. Gradually smaller subsets are selected and zoomed into and different markers and clinical features are visualized by color overlay. Interactive clustering and linked visualization of the created cluster features and statistics are demonstrated.
